# Hoarseness and Loss of Voice as Symptoms*Read before the Buffalo Medical and Surgical Association, April 7, 1885.

**Published:** 1885-05

**Authors:** F. W. Hinkel

**Affiliations:** 421 Pearl street


### BUFFALO

### Medical and Surgical Journal.

Vol. XXIV. MAY, 1885. No. 10.

### Original Communications.

### Hoarseness and Loss of Voice as Symptoms.*

By F. W. Hinkel, M. D.

It is easy to fall into a routine method of treating symptoms or groups of symptoms, especially in chronic disease. It often helps out of a dilemma, and gives an air of facility and readiness to treatment that is very effective. Particularly is this prone to occur where the causation of symptoms is obscure or difficult to analyze. Nowhere is this habit of treatment more frequent, nowhere more dangerous or disappointing, than in affections ot the larynx.

The general lack of familiarity with the mechanism of this organ, and still more with the simple apparatus necessary to examine its conditions, perhaps accounts for this, as well as for a lack of interest in this class of cases. And yet they are not without interest and importance, both on account of their frequency, and the dire results which not infrequently follow in their train.

* Read before the Buffalo Medical and Surgical Association, April 7, 1885.

I have chosen for my topic this evening the only symptoms which almost invariably are present in laryngeal affections; the only symptoms which invariably point to some involvement of the larynx—hoarseness and loss of voice. These symptoms, whatever their cause, differ little in character in different cases—only in degree. By their aid alone we are left almost entirely in the dark as to the nature of the given case presenting them, and as to the method of treatment best adapted to restore the parts to normal condition and function.

By a brief consideration of the principal diseased conditions which may produce the above symptoms, I hope to awaken more interest in the laryngoscope as a means of accurate diagnosis and treatment, and thus indirectly to renew interest in these diseases themselves.

Hoarseness and loss of voice are frequent occurrences in this climate. They deserve careful attention in almost every case. They are danger signals, which, though often raised for slight cause, yet at times give warning of approaching suffering and death, which may be avoided or averted, if heed be given in time. Of course to produce hoarseness and loss of voice there must be direct or indirect impairment of the functional integrity of the vocal bands. Anything which causes inability of the bands to vibrate with sufficient rapidity to produce sonorous vibrations of the air will cause aphonia. If the vocal bands do not vibrate in harmony with each other, and with regularity, hoarseness is produced. The causes of voice impairment vary from slight and temporary congestion to tubercular or malignant ulceration, with all their attendant horrors.

A frequent and the simplest cause of hoarseness is sub-acute congestion of the laryngeal mucous membrane. This produces slight unevenness of the edges of the bands and some infiltration of their substance. This impedes the delicate co-ordinated actions of the intra-laryngeal muscles. As a result the adduction and tension of the vocal bands are imperfect, and huskiness of tone occurs.

This same pathological condition in greater degree produces acute laryngitis. The congestion and infiltration may go on to engorgement of the tissues. The vocal bands, swollen, red and irregular are stiff with exuded serum and cell-infiltration. The muscular fibres, sodden and distended, contract with difficulty. The tumefied interarytenoid space offers a mechanical obstacle to adduction of the vocal processes. The inflamed and thickened ventricular bands encroach upon the vocal bands and impede what little vibratory power remains. The voice is reduced to a whisper and even that is produced with difficulty. Fever and general constitutional disturbance add to the discomfort of the patient. In children this is a serious condition. The small respiratory space they normally possess is greatly encroached upon. Dyspnœa and carbonic acid poisoning impend at any moment. To this mechanical obstruction in children is often added a spasmodic element, especially at night.

In debilitated subjects, or those suffering from blood poisoning in some shape, œdema of the larynx may be added to the inflammatory condition, and is frequently fatal. I but mention, in passing, as possible acute causes of aphonia or dysphonia, foreign bodies in the larynx and abscess of the larynx.

Acute and grave conditions like the above will never fail to receive your prompt attention, so I need not dwell upon them, but pass on to other forms of laryngeal disorder accompanied by impairment of voice, which, in the press of many duties, may be passed over without the investigation that their possible gravity may demand.

The most common form of laryngeal disease producing hoarseness in our climate is chronic laryngitis. It is often the result of repeated attacks of sub-acute congestion, though frequently it appears to bear its sluggish type from the first. As a corollary to this proposition let me add that it is important to give attention to the "dregs" of acute attacks and to assist the tissues to regain their lost tone, lest the seeds of this disease be left.

Chronic laryngitis is marked by the same congestion and infiltration as in acute cases, but the color is not so deep and more irregularly marked, and the infiltration tends more to the formation of connective tissue elements. The voice impairment is due to mechanical causes, as in acute cases, but the relations are different. The vocal bands are not stiffened with exudate, but relaxed and slightly thickened rather than swollen; and the innervation so affected as to produce more or less paralysis of their muscles. The interarytenoid space may, in cases marked by acute exacerbations, be the seat of shallow ulcerations or erosions whose swollen edges prevent exact approximation of the vocal processes. I have lately seen a case, with marked impairment of voice, where this tumefaction was so pronounced that on first examination I feared I had to deal with a new growth. In some cases occurs an irregularly granular condition of the vocal bands, but I am inclined to regard this form as associated with some general dyscrasia. An excess of glairy secretion is at times a cause of hoarseness in these cases, especially where the voice "breaks" and clears repeatedly during the day.

The tendency of chronic laryngitis is not toward spontaneous recovery, and the longer it exists the more difficult are its lesions to remove. Besides, it undoubtedly may lay the foundation for inflammatory involvement of the adjacent tracheal and bronchial mucous membrane, and through this for graver lesions, as catarrhal pneumonia, phthisis, pulmonary and laryngeal. With it are usually found further involvement of the pharynx and nose, which, by the way, often stand in a causal relation.

Another and grave form of disease, of which impairment of voice is a symptom, is laryngeal phthisis. I will not enter into the discussion as to whether this is always a manifestation of a local deposit of tubercle, or is of simple inflammatory origin. In pursuance of my purpose in reviewing the lesions, that may be the cause of hoarseness and loss of voice, we need not consider their pathology further than to appreciate the macroscopic appearances in a general way, and the causes of functional perversion. In this disease the first stage is indistinguishable from simple catarrhal laryngitis; or, to express it otherwise, and according to my belief, a primary simple laryngitis may, under certain local and constitutional conditions, terminate in phthisis of the larynx. The next stage of this disease is marked usually by an ashy pallor of the mucous membrane of the pharynx and larynx, and by a peculiar form of thickening and œdema that affects the aryepiglottic folds, especially about the arytænoids. This swelling is distinctly pyriform in well-marked cases, the greatest thickening being over the arytænoids. It extends into the interarytenoid space, and offers a marked mechanical obstruction to adduction of the vocal processes. This appearance of the aryepiglottic folds, when well-developed, is pathognomonic of laryngeal phthisis. It may be unilateral, or so imperfectly developed that the diagnosis must be made from the sum of the bodily conditions, or left in doubt. It is in this stage that this disease is often curable by proper local and general treatment. Hence the importance of an early recognition of this condition. Slight hoarseness may be its only marked symptom at first. Especially is this so when the lesion, at least so far as signs show it, begins in the larynx. Without a laryngoscopic examination it is impossible, under such circumstances, to differentiate this from simple laryngitis, new growth or slight paresis of the vocal muscles. Yet its gravity, and above all the urgent need of early treatment, are infinitely greater. The final stage of ulceration, with its attendant dysphagia, and often dyspnœa, are not so likely to pass without attention. Yet, I have seen cases far on in the ulcerative stage, and beyond all therapeutics except palliation, whose medical advisers seem to have been unaware that any serious disease of the larynx was in progress, no laryngoscopic examination having been made or advised. The ulcerated surfaces have a peculiar worm-eaten appearance. The ulcers are shallow, without areola and covered with a pultaceous muco-pus. If the epiglottis is involved, dysphagia is marked. The history of a case at this time is one of cruel, hopeless suffering. Anodyne and anæsthetic applications, nicely localized with the aid of the mirror, do much toward producing comparative comfort and enthanasia, and may prolong life for a considerable time.

I turn with pleasure to a not infrequent, and, since the discovery of the laryngoscope, by no means helpless class of cases producing vocal disability. I refer to new growths in the larynx. In no field has the laryngoscope greater triumphs to boast than in this. From an obscure and often fatal class of diseases they have become one of the best known, and the percentage of cures under proper surgical treatment rivals that of similar surgery of any other organ. New growths in the larynx, as elsewhere, may be divided into benign and malignant. The benign growths, fortunately, are most frequent. They are usually a result of previous inflammatory action. Their most common cause is catarrhal inflammation. I have seen cases where I believe I witnessed the inception of benign new growths which were aborted by astringent and alterative applications. Aphonia and dysphonia are the most frequent symptoms. Dysphagia may occur if growth is on epiglottis. Cough, expectoration and pain seldom occur. In benign growths there are no constitutional symptoms. Except voice disability there are often no symptoms, in early stage of development, at least. The symptoms are identical with those of chronic laryngitis. The laryngoscope alone decides the diagnosis. The most frequent form of new growths are papillomata. They are usually sessile, often multiple, of pink or grayish, irregular appearance. They may occur upon any part of the larynx, but are most frequent upon the vocal and ventricular bands. Other forms of benign growths are fibromata, usually pedunculated, and ot bright red color, angiomata and myxomata. The last two are of rare occurrence. The malignant growths are often, with difficulty, differentiated from benign growths in their early stages. There are peculiar conditions as to appearance, hardness, etc., that assist in the diagnosis, which, sooner or later, is unmistakable.

Epithelioma is the most common form of cancer. Sarcoma also occurs. Extirpation offers little or no hope, and the prognosis is a lingering and horrible death.

Another laryngeal lesion, productive of the symptoms under consideration, is paralysis of one or more of the muscles which produce the various movements of the lanynx essential to the production of tones. Laryngeal, like general paralysis, may be of central or peripheral origin. Central paralysis is rare and usually accompanied by paralysis of other muscles, and general symptoms of a character to attract attention to the nature of the disease.

Peripheral paralysis may be due to pressure or to inflammatory action. The first form is rare, except that due to pressure upon recurrent laryngeal. This is caused occasionally by aneurism, enlarged cervical glands, or even induration of apex of lung, and is almost always unilateral.

The appearances produced by complete paralysis of this nerve are diagnostic and striking. It supplies both abductor and adductor muscles, and as a result of its loss of power the affected vocal band hangs immovable midway between abduction and adduction, in the so-called cadaveric position. Its fellow approaches and passes the middle line in the effort to phonate. It remains immovable. Inspiration abducts the normal band. The position of the affected one is unchanged. Of course power of phonation is lost, and coughing, sneezing, and clearing of the throat are altered in character. A partial affection of the nerve will give paralysis either of abduction or adduction, according to filaments affected. Paralysis of the abductors, though of the greatest gravity and interest, does not immediately affect phonation, so need not fall under our consideration.

Bilateral paralysis of the adductors is a frequent form of laryngeal paralysis. On laryngoscopic inspection the glottis is seen wide open, and an effort to phonate produces only a quivering effort toward adduction. The vocal bands are abducted to their full extent, only the edges appearing under the margins of the ventricular bands. It is frequently hysteric in origin, though previous nasal catarrh, chronic laryngitis, lead poisoning, and the later stage of pulmonary phthisis, are amongst its causes. The voice is, of course, entirely lost, and during whispered speech there is shortness of breath due to the waste of expired air escaping through the wide open glottis. Unilateral paralysis of the adductors is usually the result of inflammation, either simple or specific. The laryngoscope shows one vocal band in extreme abduction. There is generally congestion ot the larynx. The voice is hoarse, husky, or entirely lost.

Another adductor paralysis is that affecting the arytenoideus. As a result of this there remains, upon attempted phonation, a triangular opening between the arytenoids, the apex being anterior. The vocal processes are approximated by the other adductors, and the ligamentous portions of the vocal bands are adjusted for phonation, but the bodies of the arytenoids are not drawn together, and through the opening thus left the air escapes, preventing sufficient vibration of the ligamentous portion to produce sound.

Lastly, the tensor muscles—the thyro-cricoid, and thyro-arytenoid—may be paralyzed. Both the bilateral and unilateral forms of thyro-cricoid paralysis are rare. As a result, tension of the vocal bands is relaxed or abolished. The voice is gruff, monotonous, or suppressed. The other tensor, the thyro-arytenoid, is often affected by inflammation of the mucous membrane of the larynx—probably on account of its superficial situation. There is, in consequence, a lack of tone about the vocal bands, and their approximation is imperfect. There is left a more or less marked space between their edges. If both muscles are affected, an elliptical space remains. If but one, the appellation "Indian-bow paralysis" expresses its appearance. These appearances above described, of course, are varied in partial or complicated paralytic conditions.

We have now briefly reviewed the principal conditions which may produce hoarseness or loss of voice. They are all a source of discomfort and uneasiness to their victims. Some of them are painful and deadly. In all of them the only positive, ever present symptom is impairment of the voice. The quality of this impairment does not differ markedly, even to a trained ear, in the different classes of causative conditions. The accompanying general bodily conditions usually give us no hint as to the true state of affairs. The cause of hoarseness may be a trifling congestion or relaxation. It may be a new growth threatening aspleyxia; or a beginning laryngeal phthisis. A diagnosis or prognosis from symptoms alone is groping in the dark. Knowledge of the exact cause and condition can be obtained by the laryngoscope alone. It turns a flood of light upon the question, and points the way to correct therapeutics.

In view of these facts it needs no long argument to demonstrate the advantage of the use of the laryngoscope in all cases when voice impairment exists. Unfortunately it is not used by the profession at large to the extent that it should be in view of the simplicity of its mechanism, and the comparative ease with which it can be employed. No complicated apparatus is essential. Bright day-light, gas, or an ordinary lamp, as a source of light, a head-reflector, and a throat mirror are sufficient. With bright day-light even a head-reflector is not a necessity. In an average case the obstacles to a laryngoscopic examination are slight. A little practice will give the co-ordination of movements requisite to steady the throat-mirror at the proper angle, and to focus upon it the reflected light. While it would be manifestly unjust to expect the general practitioner, with his multiplicity of duties and cares often of a graver sort, to be an expert in the use of this little instrument; yet, I think none will disagree that every graduate and every practitioner should have some familiarity with the methods of laryngoscopic examination. Especially is this true of those who are called upon to treat the various affections of the vocal organs which abound in this climate.

The treatment of hoarseness and loss of voice is, of course, the treatment of the causative disease. The general principles of treatment of inflammations of mucous membranes are the same here as in other parts of the body. Their application varies with the conditions and the conveniences at hand. In acute inflammations, heat, moisture and sedation, in the form of steam inhalations, external compresses, etc., are off service. As the acute stage passes off, astringent stimulants, in the form of sprays, lozenges, and occasionally powders, do good in assisting the relaxed tissues to the normal tone.

Chronic inflammations demand stimulation from the first. The brush for local applications is occasionally of service. Stimulant sprays give the best results. Perverted conditions of nose and pharynx imperatively demand attention, as there usually lie the primary cause and irritant in chronic conditions. Galvanism, externally over thyroid, is of benefit. Accurate localization of remedies and patience are two prime requisites ot success.

New growths when non-malignant almost invariably demand removal. This is best done by forceps, snare or cautery applied intra-laryngeally. When malignant, extirpation of larynx is to be thought of, but palliation is usually all that remains to be done.

In paralytic conditions, applications of faradic current, one pole over thyroid and the other upon or near the affected muscles, give good results usually, if paralysis is not of such long standing that wasting of muscular fibres has supervened. Inflammatory conditions should first be removed by topical medication to obtain best results from electricity.

Whatever be the remedies chosen, or the method of application adopted in a given case, the result will depend largely upon the accuracy of the preceding diagnosis, and the care with which the remedy is applied to the exact seat of disease.

421 Pearl street.

